# Supplementary material for: Caspase-11 promotes allergic airway inflammation
Source: Nat Commun. 2020 Feb 26;11:1055. doi: 10.1038/s41467-020-14945-2 (PMC7044193; doi:10.1038/s41467-020-14945-2)
Supplement: Supplementary file 1 — Supplementary Information [file 41467_2020_14945_MOESM1_ESM.pdf]

Supplementary Data

Caspase-11 promotes allergic airway inflammation

[1#]Zbigniew Zasłona, [1]Ewelina Flis, [1]Mieszko M. Wilk, [1]Richard G. Carroll, [1]Eva M. Palsson-McDermott, [1]Mark M. Hughes, [1]Ciana Diskin, [1]Kathy Banahan, [1]Dylan G. Ryan, [1]Alexander Hooftman, [1]Alicja Misiak, [1]Jay Kearney, [2]Gunter Lochnit, [3]Wilhelm Bertrams, [4]Timm Greulich, [3,4]Bernd Schmeck, [5]Oliver J. McElvaney, [1]Kingston H.G. Mills, [1]Ed C. Lavelle, [2]Małgorzata Wygrecka, [1]Emma M. Creagh, [1]Luke A.J. O'Neill

[1]School of Biochemistry and Immunology, Trinity Biomedical Sciences Institute (TBSI), Trinity College Dublin, Ireland
[2]Department of Biochemistry, Faculty of Medicine, Justus Liebig University, Giessen, Germany
[3]Institute for Lung Research, Universities of Giessen and Marburg Lung Center, Philipps-University Marburg, Member of the German Center for Lung Research (DZL), Marburg, Germany
[4]Department of Medicine, Pulmonary and Critical Care Medicine, University Medical Center Giessen and Marburg, Philipps-University, Member of the German Center for Lung Research (DZL), Marburg, Germany
[5]Royal College of Surgeons in Ireland, Beaumont Hospital, Dublin, Ireland

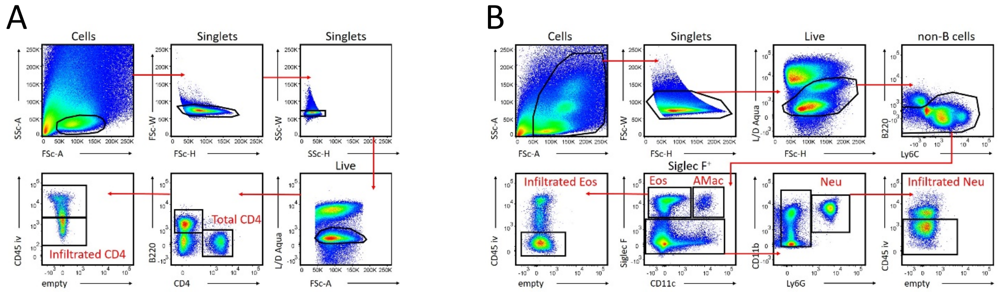

Supplementary Figure 1. Gating strategies used in Fig. 4 to determine infiltration of immune cells into the lungs.

To discriminate blood-borne circulating cells from lung infiltrated cells, we used CD45 i.v. administration 10 min before mouse were sacrificed and lungs were harvested. Gating strategies used to determine: **(A)** infiltrated CD4 T cells and **(B)** infiltrated eosinophils and neutrophils.

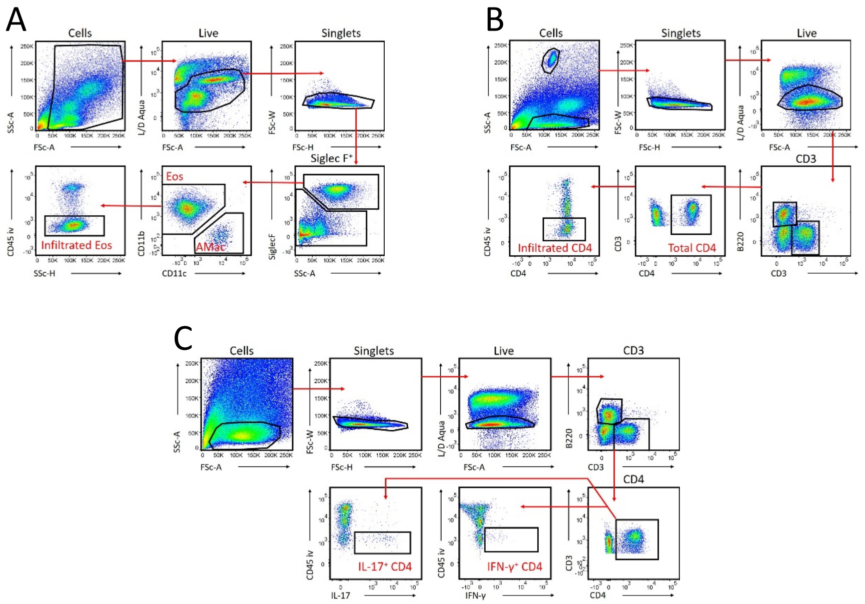

Supplementary Figure 2. Gating strategies used in Fig. 5 to determine infiltration of immune cells into the lungs.

To discriminate blood-borne circulating cells from lung infiltrated cells, we used CD45 i.v. administration 10 min before mouse were sacrificed and lungs were harvested. Gating strategies used to determine: **(A)** infiltrated eosinophils **(B)** infiltrated CD4 T cells and **(C)** cytokine secreted infiltrating CD4 T cells.
