# Supplementary material for: Caspase-11 promotes allergic airway inflammation
Source: Nat Commun. 2020 Feb 26;11:1055. doi: 10.1038/s41467-020-14945-2 (PMC7044193; doi:10.1038/s41467-020-14945-2)
Supplement: Supplementary file 3 — Reporting Summary [file 41467_2020_14945_MOESM3_ESM.pdf]

nature research

Corresponding author(s): Zbigniew Zaslona

Last updated by author(s): Feb 5, 2020

Reporting Summary

Nature Research wishes to improve the reproducibility of the work that we publish. This form provides structure for consistency and transparency in reporting. For further information on Nature Research policies, see Authors & Referees and the Editorial Policy Checklist.

Statistics

For all statistical analyses, confirm that the following items are present in the figure legend, table legend, main text, or Methods section.

n/a	Confirmed	
☐	☒	The exact sample size (*n*) for each experimental group/condition, given as a discrete number and unit of measurement
☐	☒	A statement on whether measurements were taken from distinct samples or whether the same sample was measured repeatedly
☐	☒	The statistical test(s) used AND whether they are one- or two-sided<br>*Only common tests should be described solely by name; describe more complex techniques in the Methods section.*
☐	☒	A description of all covariates tested
☐	☒	A description of any assumptions or corrections, such as tests of normality and adjustment for multiple comparisons
☐	☒	A full description of the statistical parameters including central tendency (e.g. means) or other basic estimates (e.g. regression coefficient) AND variation (e.g. standard deviation) or associated estimates of uncertainty (e.g. confidence intervals)
☒	☐	For null hypothesis testing, the test statistic (e.g. *F*, *t*, *r*) with confidence intervals, effect sizes, degrees of freedom and *P* value noted<br>*Give P values as exact values whenever suitable.*
☒	☐	For Bayesian analysis, information on the choice of priors and Markov chain Monte Carlo settings
☒	☐	For hierarchical and complex designs, identification of the appropriate level for tests and full reporting of outcomes
☒	☐	Estimates of effect sizes (e.g. Cohen's *d*, Pearson's *r*), indicating how they were calculated

Our web collection on statistics for biologists contains articles on many of the points above.

Software and code

Policy information about availability of computer code

Data collection	ABI 7500 Fast real-time PCR system (Applied Biosystem) - qPCR<br>ChemiDoc imaging system (Bio-Rad), X-ray films - Western blott<br>Fluostar OPTIMA software data analysis (BMG Labtech) - LDH assay, ELISA<br>FlowJo LLc - flow cytometry
Data analysis	Graph Pad Prism

For manuscripts utilizing custom algorithms or software that are central to the research but not yet described in published literature, software must be made available to editors/reviewers. We strongly encourage code deposition in a community repository (e.g. GitHub). See the Nature Research guidelines for submitting code & software for further information.

Data

Policy information about availability of data

All manuscripts must include a data availability statement. This statement should provide the following information, where applicable:

- Accession codes, unique identifiers, or web links for publicly available datasets
- A list of figures that have associated raw data
- A description of any restrictions on data availability

Any further data not included in the manuscript is available from the corresponding author on reasonable request.

Field-specific reporting

Please select the one below that is the best fit for your research. If you are not sure, read the appropriate sections before making your selection.

☒ Life sciences ☐ Behavioural & social sciences ☐ Ecological, evolutionary & environmental sciences

For a reference copy of the document with all sections, see nature.com/documents/nr-reporting-summary-flat.pdf

Life sciences study design

All studies must disclose on these points even when the disclosure is negative.

Sample size	For in vitro studies we have used 3 biological replicates in each experiment, unless differently specified in figure description. This is designed to account for biological variability since experiments were performed in primary murine macrophages.<br>Sample size for in vitro caspase-11 dependent pyroptosis measurements was determined based on a previous study examining this process using the same reagents and protocol Nature. 2015 Oct 29;526(7575):666-71.<br>For in vivo allergic airway inflammation sample size determination we have used a previous study, which utilized the same protocol, the same mouse strain, age and sex and the same dose of Misoprostol treatment. J Allergy Clin Immunol. 2014 Feb;133(2):379-87
Data exclusions	In the source data file we have shown excluded data for flow cytometry analysis. Data was excluded from a mouse, where allergic airway disease was not induced based on leukocyte infiltration to the lung, which is our readout of diseases induction (Figure 5d-f).
Replication	Presented results are highly reproducible and were replicated, experiments performed once are refereed to in manuscript as preliminary data. (Fig. 1d and Figure 2d).
Randomization	Mice were randomly assigned to different treatment groups in our study.
Blinding	In vivo allergic airway inflammation studies were blinded.

Reporting for specific materials, systems and methods

We require information from authors about some types of materials, experimental systems and methods used in many studies. Here, indicate whether each material, system or method listed is relevant to your study. If you are not sure if a list item applies to your research, read the appropriate section before selecting a response.

Materials & experimental systems

n/a	Involved in the study	
☐	☒	Antibodies
☒	☐	Eukaryotic cell lines
☒	☐	Palaeontology
☐	☒	Animals and other organisms
☐	☒	Human research participants
☒	☐	Clinical data

Methods

n/a	Involved in the study	
☒	☐	ChIP-seq
☐	☒	Flow cytometry
☒	☐	MRI-based neuroimaging

Antibodies

Antibodies used	β-actin (1:15,000, AC-74; Sigma-Aldrich), Gapdh (1:5000, 6C5, Calbiochem), IL-1β (pro- and cleaved; 1:1000, AF-401; R&D Systems), caspase-11 (1:1000, 17D9, Sigma-Aldrich), p-STAT1 (1:1000, #9177, Ser727, Cell Signalling) and total STAT1 (1:1000, #9172, Cell Signalling). HRP-conjugated secondary Abs were from Jackson ImmunoResearch Laboratories.
Validation	All above antibodies have been used in multiple papers by our and other groups.

Animals and other organisms

Policy information about studies involving animals; ARRIVE guidelines recommended for reporting animal research

Laboratory animals	Experiments were performed with 8-to 12-wk-old female mice bred under specific pathogen-free conditions,
Wild animals	na
Field-collected samples	na

Ethics oversight | All experiments were carried out with prior ethical approval from the Trinity College Dublin Animal Research Ethics Committee.

Note that full information on the approval of the study protocol must also be provided in the manuscript.

Human research participants

Policy information about studies involving human research participants

Population characteristics
Asthma was diagnosed by symptoms in conjunction with airway hyperresponsiveness (metacholine challenge). Furthermore, the concentration of nitrogen monoxide (FeNO) for all patients and controls was measured.

Recruitment
Patients with clinically confirmed bronchial asthma and healthy volunteers were recruited by the Clinic for Internal Medicine - Department for Pneumology, University Medical Center Marburg. Human subject participating in this study signed informed consent to participate in this research.

Ethics oversight
BALF was obtained following ATS consensus procedure in accordance with local ethics regulations (87/12) from healthy subjects and asthma patients. Study was approved and oversaw by Ethics Committee of the Medical Faculty of the Philipps University, Marburg, Germany.

Note that full information on the approval of the study protocol must also be provided in the manuscript.

Flow Cytometry

Plots

Confirm that:

☒ The axis labels state the marker and fluorochrome used (e.g. CD4-FITC).

☒ The axis scales are clearly visible. Include numbers along axes only for bottom left plot of group (a 'group' is an analysis of identical markers).

☒ All plots are contour plots with outliers or pseudocolor plots.

☒ A numerical value for number of cells or percentage (with statistics) is provided.

Methodology

Sample preparation
Lung tissue was chopped and digested with collagenase D (1 mg/ml; Roche) and DNase I (10 µg/ml; Sigma-Aldrich) for 1 h at 37°C with agitation. Next, lungs or spleens were passed through a 40-µm cell strainer to a obtain single-cell suspension, followed by RBC lysis. The cells were incubated with CD16/CD32 FcγRIII (1:100) to block IgG Fc receptors. Cells were incubated with LIVE/DEAD Aqua (Invitrogen), followed by surface staining with fluorochrome-conjugated anti-mouse Abs for various markers. To detect cytokines, cells were stimulated with PMA (50 ng/ml) and ionomycin (500 ng/ml) in the presence of brefeldin A (5 µg/ml) for 4 h at 37°C. For detection of intracellular cytokines, cells were fixed in 2% PFA and permeabilized with 0.5% saponin (Sigma-Aldrich, Ireland), followed by staining with IL-17A–V450 and IFN-γ–PE–CF594 (BD Biosciences). To discriminate blood-borne circulating cells from lung-localized cells, we used a well-described approach in which anti-mouse PE-CD45 Ab (eBioscience) was administered i.v. to mice 10 min before they were euthanized and lungs were harvested. Circulating leukocytes, that are exposed to the antibody and are labelled, become CD45iv+, whereas tissue infiltrated cells are "protected" from labelling and remain CD45iv-.

Instrument
BD LSRFortessa™

Software
FlowJO

Cell population abundance
All measured cell populations, namely CD4 T cells, eosinophils and neutrophils are very well defined and abundant as evidenced by our gating strategy in source file data and figures 4 and 5. For measurements at least 1 million events were recorded.

Gating strategy
Gating strategy is provided in data source files and can be put in supplementary material if needed.

☒ Tick this box to confirm that a figure exemplifying the gating strategy is provided in the Supplementary Information.
